# Quantum Strategic Organizational Design: Alignment in Industry 4.0 Complex-Networked Cyber-Physical Lean Management Systems

**DOI:** 10.3390/s20205856

**Published:** 2020-10-16

**Authors:** Javier Villalba-Diez, Xiaochen Zheng

**Affiliations:** 1 Fakultät Management und Vertrieb, Campus Schwäbisch Hall, Hochschule Heilbronn, 74523 Schwäbisch Hall, Germany; 2 Complex Systems Group, Escuela Técnica Superior de Ingenieros Agrónomos, Universidad Politécnica de Madrid, Av. Puerta de Hierro 2, 28040 Madrid, Spain; 3 ICT for Sustainable Manufacturing, SCI-STI-DK, École Polytechnique Fédérale de Lausanne (EPFL), 1015 Lausanne, Switzerland; xiaochen.zheng@epfl.ch

**Keywords:** quantum computing, strategic organizational design, Industry 4.0, complex networks, cyber-physical systems, lean management systems

## Abstract

The strategic design of organizations in an environment where complexity is constantly increasing, as in the cyber-physical systems typical of Industry 4.0, is a process full of uncertainties. Leaders are forced to make decisions that affect other organizational units without being sure that their decisions are the right ones. Previously to this work, genetic algorithms were able to calculate the state of alignment of industrial processes that were measured through certain key performance indicators (KPIs) to ensure that the leaders of the Industry 4.0 make decisions that are aligned with the strategic objectives of the organization. However, the computational cost of these algorithms increases exponentially with the number of KPIs. That is why this work makes use of the principles of quantum computing to present the strategic design of organizations from a novel point of view: Quantum Strategic Organizational Design (QSOD). The effectiveness of the application of these principles is shown with a real case study, in which the computing time is reduced from hundreds of hours to seconds. This has very powerful practical applications for industry leaders, since, with this new approach, they can potentially allow a better understanding of the complex processes underlying the strategic design of organizations and, above all, make decisions in real-time.

## 1. Introduction

The most significant aspect of strategic planning in an organization is, according to Grant [1], the strategic process: “a dialog through which knowledge is shared and the consensus is achieved and commitment towards action and results is built”. To unify common efforts and, hence, support the strategic organizational goals, it is during this dialogue, previously described as Nemawashi [2] or “catch-ball” [3] by scholars, that a sometimes delicate balance of forces is sought between the interests of different organizational agents [4]. Under a strategic organizational design paradigm [5,6], the interaction of these interdependent organizational agents shapes hierarchically nested complex networks [7] that support decision making towards, ideally, a coordinated effort to attain organizational strategic goal achievement, called organizational alignment. These alignment efforts can occur in different organizational settings, although, in this paper the authors focus on complex networked cyber-physical systems in an Industry 4.0 context.

**Figure 1:**
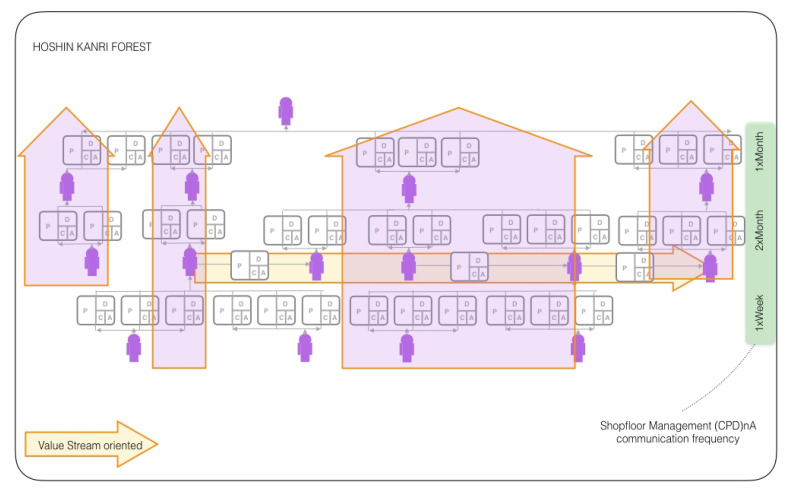
Hoshin Kanri Forest Structure [11].

For any given time *t*, complex cyber-physical networks have been formally described [8] as time-dependent graphs given by Equation (1):

(1) Ω(t)=[Γ(t);E(t)]

which can be understood as lists of Γ(t) human and cyber-physical nodes and its standard communication E(t)⊂(Γ(t)xΓ(t)) edges [9]. The very emergence of complex networked organizational design configurations in the form of lean structural networks is only possible through a continuous improvement-oriented standardization of the organizational network edges, the business communication protocols, between the network elements [9]. These boundary conditions allow for representing the systems of Industry 4.0 as cyber-phsycial complex networks, allowing a systematic and quantitative analysis of the systems by means of lean management algorithms strategically oriented to the systematic reduction of the variability of the value creation processes. For this reason, this work is focused solely on lean management systems in an Industry 4.0 cyber-physical context. The standard repetition of *Check, Plan, Do* n-times—with a subsequent process standardization in *Act*, in short *(CPD)nA*, allows for a comprehensive management of such networks through HOSHIN KANRI FOREST [10] configurations, as shown in Figure 1.

Scholars have proposed approaches to qualitatively model organizational alignment [12,13,14,15,16,17,18,19,20]. Approaches that allow for a quantification of organizational alignment are less common [2], which shows the alignment state of each node is known at each discrete time interval. However, the NEMAWASHI approach, based on genetic algorithms, is computationally very costly and, therefore, difficult to implement in practice. While calculating the alignment state of the entire network is theoretically possible with this method, in practice, it is a challenge that leads to an exponential increase of calculation time with augmenting network size. For this reason, there is an urgent need to provide organizational leaders with a fast algorithm that allows for a calculation of the alignment status of the organization.

Quantum computing is a novel computation paradigm that might prove useful to this end [21]. Quantum computing examines the flow and processing of information as physical phenomena that follow the laws of quantum mechanics. This is possible, because quantum computing makes use of “superposition”, which is the ability of quantum computers to be simultaneously in multiple different states [22]. By doing so, quantum computing has shown promising performance increases in solving certain unassailable problems for classic computing, such as Shor’s algorithm [23] and Grover’s algorithm [24]. The purpose of this work is to propose an efficient quantum computation algorithm that is capable of discerning the organizational state of alignment of such complex networks and, therefore, support the leaders of organizations in their decision-making process.

To do so, we start with an initial hypothesis formulation. The intrinsically interdependent nature of lean complex cyber-physical networks allows for us to reasonably establish the initial hypothesis H of this work: the decision-making associated with the alignment process lacks absolute certainty.

In other words, leading an organization toward the coordinated achievement of strategic objectives is a probabilistic process, in which decision-makers can never be certain that the choice being made is the correct one. Decision-makers are conditioned by other organizational agents’ simultaneous decisions whose consequences cannot be completely foreseen a-priori. Consequently, these networks can be considered as decision networks or probabilistic directed acyclic graphical models [25] with known conditional probabilities of alignment.

The rest of the work hereinafter continues, as follows: first, Section 2 begins by defining some essential preliminary concepts for the precise use of terminology in the following sections of this work. Second, Section 3 presents the main contribution of this work by outlining the methodology and design principles proposed by this work to achieve a quantum formulation of strategic organizational design (QSOD). Third, Section 4 presents a case study to show the implementation of the theoretical design principles explained above. This is intended to allow for a better understanding and make the benefits that the application of this methodology may have for the leaders of the Industry 4.0 more explicit. Finally, Section 5 briefly summarizes the results that were obtained, as well as showing future lines of research and certain limitations to the work.

## 2. Background

This section starts by defining some preliminary concepts fundamental to the precise terminology use of the content presented in the following sections of this work:

- Industry 4.0. Industry 4.0 has gained a lot of attention since it was first released [26], claiming the necessity of a new paradigm shift in favour of a less centralized manufacture structure. It is regarded as the fourth industrial revolution, the first three being mechanization through the use of vapor energy, mass production through electricity generation, and ultimately the digital revolution through the integration of electronics and information technology. The industry 4.0 ought to allow for a larger independence of the manufacturing process, since technology is more interrelated and the machines can interact with each other creating a cyber-physical system [27,28,29,30,31,32].
- Cyber-Physical Systems. Cyber-physical system in the context of Industry 4.0 relates to the close bonding and alignment between computing and material resources. A new paradigm of technological systems that is based on embedded collaborative software is impacting the development of such systems [33,34,35].
- Lean Management. Lean management systems in a cyber-physical environment of Industry 4.0 are described as socio-technical structures that are designed to consistently reduce the variability of value creating processes and, therefore, increase their effectiveness and profitability [9,36,37,38,39,40,41,42,43,44,45,46].
  Variability in this context is understood as any deviation from the desired process state. In quantifiable terms, this work understands the variability of a process, as measured by the systematic reduction of the standard deviation that is associated with the indicators measuring its performance [9,47].
- Complex Networked Organizational Design. According to the network organizational paradigm, modern cyber-physical systems that are oriented to the lean management of Industry 4.0 can be seen as a socio-technical symbiotic ecosystem of human networks [5] interacting with distributed physical sensors interconnected in an increasingly complex network interconnected sensors [48], which readings are modeled as time-dependent signals at the vertices, human, or cyber-physical, respectively. That means that in the structure of the network nodes you can find characteristics that represent them in the form of a certain time series that describes the key performance indicators (KPIs).

On the basis of the previous concepts, the lean management of complex cyber-physical systems networked in an Industry 4.0 context, may be defined as business systems that seek systematically to decrease the inherent variability of industrial value creation processes, considering them to be complex networks of interdependent computational and physical elements. Effective and efficient calculation of the information that flows through these elements is the key factor for achieving lasting and sustained business success.

- Alignment. This information is typically described by a series of KPIs. Such KPIs are interdependent and they describe certain trajectories in node-related orthonormal bases [2]. These scholars define a node to be in alignment at any given moment in time if the KPI’s trajectory presents asymptotic stability at this point [49]. In other words, the condition for alignment at any given time interval *t+Δt* is given by Equation (2)
  (2) $$\forall \Delta t>0\;D_{t,t+\Delta t}<D_{t-\Delta t,t}$$
  where $D_{i,j}$ represents the euclidean distance between two points *i* and *j* in the KPI’s trajectory. Consequently, the probability that the node is not in alignment is given by Equation (3)
  (3) $$\forall \Delta t>0\;D_{t,t+\Delta t}\geq D_{t-\Delta t,t}$$
  where $D_{i,j}$ represents the euclidean distance between two points *i* and *j* in the KPI’s trajectory. Thus, alignment is a binary property of each node. Furthermore, since the trajectories are known ∀t, we can calculate the conditional probability that the nodes within the complex networked SOD are simultaneously in alignment or not, by the simple application of the well known Bayes theorems.
  In fact, within this time interval Δt, the graph Ω that is described in Equation (1) converts into a decision network $\Omega^{\prime }=\left[\Gamma^{\prime },E^{\prime }\right]$ formed by a set of $\Gamma^{\prime }$ nodes and $E^{\prime }$ edges, where $\Gamma^{\prime }=\left[\gamma_{1},\gamma_{2},\ldots ,\gamma_{N}\right]$ represents the set of all the nodes being part of the network in Δt, and the edges are determined by the known probabilistic dependence of alignment occurrence in a node $\gamma_{j}$, depending on the alignment occurrence on another $\gamma_{i}$. The node $\gamma_{i}$ is thus called *parent* and node $\gamma_{j}$ the child. The root nodes are those that do not depend on any other. Subsequently, as described by [50], the joint probability on the nodes can be decomposed into the product of the marginal probabilities that are given by Equation (4):
  (4) $$P\left(\gamma_{1},\gamma_{2},\ldots ,\gamma_{N}\right)=\prod_{i=1}^{N}P\left(\gamma_{i}|\prod \gamma_{i}\right)$$
  where $\prod \gamma_{i}$ represents the set of parent nodes associated with $\gamma_{i}$. For the root nodes, $P(\gamma_{i}|\prod \gamma_{i})$ becomes the marginal distribution $P(\gamma_{i})$. This property shall be used later on for a proper representation of lean complex cyber-physical networks through quantum circuits.

The following paragraphs present several quantum computing fundamentals for the general reader.

**Figure 2:**
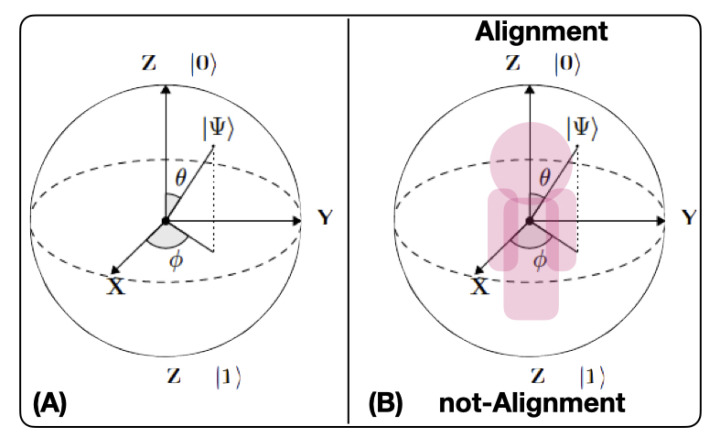
(**A**) Bloch sphere (**B**) Quantum Strategic Organizational Design (QSOD) Bloch sphere.

- Qubit
  Information may be represented in many different ways. Quantum computing uses quantum discrete units of information, the qubit (quantum bit) [51]. Qubits represent elementary units of information exchange in quantum computing, similar to the “bits” of classical computing. A bit is always in two basic states, 0 and 1, while a qubit can be in both bases of these states simultaneously. The characteristic is also known as superposition. Quantum computing normally uses the Dirac notation that represents the two bases of computing of these states |0〉 and |1〉. The superposition of a |Ψ〉 qubit is merely a linear combination of the two basic states |0〉 and |1〉, expressed by the Equation (5):
  (5) $$|\Psi \rangle =c_{1}|0\rangle +c_{2}|1\rangle$$
  where $c_{1}$ and $c_{2}$∈C such that the satisfy the Equation (6):
  (6) $${\left|c_{1}\right|}^{2}+{\left|c_{2}\right|}^{2}=1$$
  in which ${\left|c_{1}\right|}^{2}$ and ${\left|c_{2}\right|}^{2}$ are, respectively, the probabilities of finding the *qubit* in |0〉 and |1〉 after a measurement in the (|0〉,|1〉) basis.
- Bloch’s sphere
  Bloch’s sphere, as shown in Figure 2A, is commonly used to geometrically represent a *qubit* [52]. This is a useful and common geometric image of the quantum evolution of a single- or two-level system. On the Bloch sphere, of unitary radius, the *Z*-axis is the computational axis and its positive direction coincides with the state |0〉, and the negative with the state |1〉. A *qubit* can be represented as a point on the Bloch sphere with the help of two parameters (θ, ϕ), as expressed by Equation (7):
  (7) $$|\Psi \rangle =cos\left(\frac{\theta }{2}\right)|0\rangle +e^{i\phi }sin\left(\frac{\theta }{2}\right)|1\rangle$$
  When several qubits are utilized, their aggregated state can be determined utilizing the tensorial product of the individual qubits. If the multiple qubit state can be expressed as a linear combination of the |0〉 and |1〉 states, then the aggregated state can be represented, as in Equation (8):
  (8) $$|\Psi_{1}\rangle ⊗|\Psi_{2}\rangle =c_{11}c_{21}|00\rangle +c_{11}c_{22}|01\rangle +c_{12}c_{21}|10\rangle +c_{12}c_{22}|11\rangle$$
  where $|\Psi_{1}\rangle =c_{11}|0\rangle +c_{12}|1\rangle$ and $|\Psi_{2}\rangle =c_{21}|0\rangle +c_{22}|1\rangle$. However, if the aggregate state cannot be expressed as the product of the individual states, in other words, if no qubit states |a〉 and |b〉 can be found, such that |Ψ〉=|a〉|b〉, this state is called entangled state, which is stronger than any other classical correlation [53].
  The reduced purity $\kappa_{j}$ of a qubit $q_{j}$ in an N−qubit state |Psi〉, as given by Equation (9), is a coefficient $\kappa_{j}\in \left[0.5,1\right]$ that indicates the level of a qubit entanglement in the state [54]. A value of $\kappa_{j}=1$ indicates that the qubit is not entangled with the other N−1 qubits, and a value of $\kappa_{j}=0.5$ indicates that the qubit is maximally entangled with the other qubits in the state.
  (9) $$\kappa_{j}=Tr{\left[Tr_{i\in [0,N-1],i\neq j}\;|\psi \rangle \langle \psi |\right]}^{2}$$
- Quantum circuit
  A quantum circuit is a computational sequence that consists of performing a series of coherent quantum operations on qubits. By organizing the qubits into an orderly sequence of quantum gates, measurements, and resets, all of which can be conditioned and use data from the classical calculation in real-time, quantum computing can be simulated. These sequences typically follow a standardized pattern:
  1. Initialization and reset. First, we begin our quantum calculation with a specified quantum state for each qubit. This is achieved using the initialization operations, typically on the Z-computation axis, and reset. The resets can be done using a single-qubit gate combination that tracks whether we have succeeded in creating the desired state through measurements. Qubit initialization in a desired state |Ψ〉 can then continue to apply single-qubit gates.
  2. Quantum gates. Second, we implement a sequence of quantum gates that manipulate the qubits, as needed by the targeted algorithm following certain quantum circuit design principles.
  3. Measurement. Third, we measure the qubits. Classical computers translate the measurements of each qubits as classical results (0 and 1) and then store them in either one of the two classical bits. Measurement is understood to be projected into the *Z*-computational basis unless otherwise stated.
- Quantum gate
  A quantum gate consists of several mathematical operations applied to the qubits that change the amplitude of their probabilities and, thus, perform the intended computations [54]. The quantum computing basic elements are described in detail:
  – The $U_{3}\left(\theta ,\phi ,\lambda \right)$ gate is a single qubit gate that has three parameters θ, ϕ and λ which represent a sequence of rotations around the Bloch sphere’s axes such that [ϕ−π/2] around the *Z* axis, [π/2] around the *X* axis, [π−θ] around the *Z* axis, [π/2] around the *X* axis, and a [λ−π/2] around the *Z* axis. It can be used to obtain any single qubit gate. Equation (10) provides its mathematical representation,
    (10) $$U_{3}|\Psi \rangle =\left[\begin{array}{cc} cos(\frac{\theta }{2}) & -e^{i\lambda }sin\left(\frac{\theta }{2}\right)\\ e^{i\phi }sin\left(\frac{\theta }{2}\right) & e^{i(\phi +\lambda )}cos\left(\frac{\theta }{2}\right) \end{array}\right]|\Psi \rangle$$
    and Equation (11) its quantum circuit equivalent:

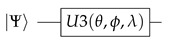
(11)
  – The CNOT or conditional NOT gate is a two qubit computation gate with one qubit acting as control $|\Psi_{1}\rangle$ and the other as *target*$|\Psi_{2}\rangle$. The CNOT gate performs a selective negation of the target qubit. If the control qubit is in superposition, then CNOT creates entanglement. Equation (12) provides its mathematical representation,
    (12) $$CNOT|\Psi_{1}\rangle |\Psi_{2}\rangle =|\Psi_{1}\rangle |\Psi_{1}⊕\Psi 2\rangle$$
    and Equation (13) its quantum circuit equivalent:

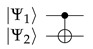
(13)
  – The ccX or Toffoli gate is a three qubit computation gate with two qubits $|\Psi_{1}\rangle$ and $|\Psi_{2}\rangle$ acting as controls and one qubit $|\Psi_{3}\rangle$ acting as target. The ccX gate applies an *X* to the target qubit $|\Psi_{3}\rangle$ only when both controls $|\Psi_{1}\rangle$ and $|\Psi_{2}\rangle$ qubits are in state |1〉. Equation (14) provides its mathematical represetnation:
    (14) $$ccX|\Psi_{1}\rangle |\Psi_{2}\rangle |\Psi_{3}\rangle =|\Psi_{1}\rangle |\Psi_{1}⊕\Psi 2\rangle |\Psi_{1}\rangle |\Psi_{1}⊕\Psi 3\rangle$$
    and Equation (15) its quantum circuit equivalent [54]:

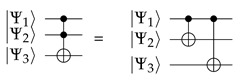
(15)
  – The *Z-measurement* of a quantum state—a self-adjoint operator on the Hilbert space—results in the measured object being in an eigenstate of the *Z* operator or computational basis, with the corresponding eigenvalue being the value measured. The measurement, also called observation, of a quantum state, is a stochastic non-reversible operation and, therefore, cannot be considered as a quantum gate, as it allocates a unique value to the variable observed. In mathematical terms, the probability *p* of a measurement result *m* occurring when the state |Ψ〉 is measured is given by Equation (16):
    (16) $$p(m)=\langle \Psi |M_{m}^{†}M_{m}|\Psi \rangle$$
    where $\left[M_{m}\right]$ represents a set of operators acting on the state space, such that $I=\sum_{m}p\left(m\right)$ and the state of the system after the measurement $|\Psi^{\prime }\rangle$ is given by Equation (17):
    (17) $$|\Psi^{\prime }\rangle =\frac{M_{m}|\Psi \rangle }{\sqrt{p(m)}}$$
    Equation (18) shows its quantum circuit equivalent as a symbolic box:

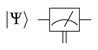
(18)

## 3. Quantum Strategic Organizational Design

This chapter outlines the main contribution of this work, presenting a model and design principles for enabling organizational leaders to perform quantum strategic organizational design. This model consists of three steps: first, it introduces the definition of the QSOD qubit as a fractal unit of the decision Industry 4.0 complex-networked cyber-physical lean management systems. Second, it outlines the design principles that are to be applied in order to represent such systems as a quantum circuit. Finally, it provides guidelines for the interpretation of the results.

### 3.1. QSOD *Qubit*

To be able to create a quantum circuit that allows for contemplating decision Industry 4.0 complex-networked cyber-physical lean management systems it is necessary, to provide a standardized vision of the elements of this network. As mentioned above, scholars have previously defined a communication standard (CPD)nA that forms the edges of these complex networks [9]. Now, it is time to present a standard for the nodes.

As depicted in Figure 2B, we define the QSOD qubit, the node of a decision complex-networked cyber-physical lean management system, as a human or cyber-physical asset that is in the center of an imaginary Bloch sphere, in which the state of alignment and not-alignment references, respectively, with the QSOD qubit |0〉 and |1〉 computational states. This is possible because the QSOD qubit exchanges information with other QSOD qubits through standardized information flow behavioral (CPD)nA patterns that allow for constant monitoring of its performance and this implies, as shown in [2], that the state of individual alignment of all nodes is known at any point in time.

### 3.2. QSOD Design Principles

- Calculation of conditional probabilities
  The conditional probabilities that will give rise to the quantum circuit are derived from a preliminary analysis of the KPIs that are associated with each node of the complex network in question. This analysis, as indicated above, is based on the method based on genetic algorithms presented in [2]. Specifically, for each node, there are typically three related KPIs. The selected chromosome has subsequently 12 real numbers between 0 and 1, and we have used real value crossover and mutation with probabilities of 60% and 7%, respectively. The population was built over 8000 individuals and it ran over 1000 generations. Once the trajectories associated with each node have been calculated, by applying the Bayes theorem, it is trivial to calculate the relative probability of alignment or non-alignment at each node concerning those to which it is connected.
- Initialization and reset
  The initialization and reset of the qubits is typically standardized to the state |0〉 on the computational *Z*-axis.
- Rotation angle computation
  The conditional probabilities translate into qubit rotation angles depending on its decision network dependencies:
  – For a root node with no parents, the possible states are two |0〉 and |1〉. A trivial application of Equation (7), states that a qubit intialized to state |0〉 and rotated by a gate U3(θ,0,0), being ϕ=0, transforms it into $|\Psi \rangle =cos\left(\frac{\theta }{2}\right)|0\rangle +sin\left(\frac{\theta }{2}\right)|1\rangle$. Therefore, taking Equation (5) into account and the definition of the Bloch sphere’s angles, the rotation angle θ that is required to calculate the probabilities of being in state |0〉 and |1〉 can be expressed by Equation (19):
    (19) $$\theta =2atan\left[tan\left(\frac{\theta }{2}\right)\right]=2atan\sqrt{\frac{sin^{2}\left(\frac{\theta }{2}\right)}{cos^{2}\left(\frac{\theta }{2}\right)}}\overset{Equation\;(5)}{=}2atan\sqrt{\frac{p(|1\rangle )}{p(|0\rangle )}}$$
  – In general, for a child node $\gamma_{i}$ with m parents, there are $2^{m}$ possible states $\prod \gamma_{i}^{*}$. Subsequently, taking Equations (4), (5), and (7) into account, as well as the definition of the Bloch sphere’s angles, the rotation angle is given by Equation (20):
    (20) $$\theta_{\gamma_{i},\prod \gamma_{i}^{*}}\overset{Equation\;(4)-Equation\;(5)}{=}2atan\sqrt{\frac{p(|1\rangle |\prod \gamma_{i}=\prod \gamma_{i}^{*})}{p(|0\rangle |\prod \gamma_{i}=\prod \gamma_{i}^{*})}}$$
- Controlled rotations
  Controlled rotations are not elementary quantum gates and they need to be deconstructed into elementary operations. As described by Nielsen and Chuang [54], being *m* the maximum number of parent nodes a child has, the controlled rotation expressing the conditional probabilities needs of the addition of $a_{i}$ “dummy” qubits i=1,…,m−1 in order to decompose the controlled rotation into 2(m−1)CNOT gates and one U3(θ,0,0). This is exemplified in Equation (21) for m=5 qubits, $a_{i}$ “dummy” qubits i=1,…,(m−1=4) and a total of 2(m−1)=8CNOT gates.

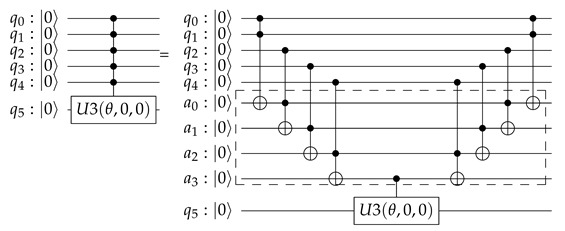
(21)
  As first shown in [55], it is important to highlight that U3(θ,0,0) is best decomposed as by Equation (22):
  (22) $$\begin{array}{c} U3\left(\theta ,0,0\right)|\Psi_{1}\rangle |\Psi_{2}\rangle =U3\left(\frac{\theta }{2},0,0\right)|\Psi_{2}\rangle CNOT|\Psi_{1}\rangle |\Psi_{2}\rangle U3\left(\frac{-\theta }{2},0,0\right)|\Psi_{2}\rangle CNOT|\Psi_{1}\rangle |\Psi_{2}\rangle \end{array}$$
  Additionally, as a direct application of Equation (13), this equation converts into Equation (23):
  (23) $$\begin{array}{c} U3\left(\theta ,0,0\right)|\Psi_{1}\rangle |\Psi_{2}\rangle \overset{Equation\;(13)}{=}U3\left(\frac{\theta }{2},0,0\right)|\Psi_{2}\rangle |\Psi_{1}\rangle |\Psi_{1}⊕\Psi 2\rangle U3\left(\frac{-\theta }{2},0,0\right)|\Psi_{2}\rangle |\Psi_{1}\rangle |\Psi_{1}⊕\Psi 2\rangle \end{array}$$
  or in its quantum circuit equivalent that is shown in Equation (24):

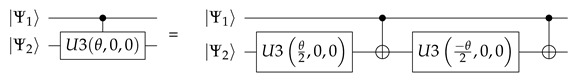
(24)
  This method works, because, if the control qubit $|\Psi_{1}\rangle$ is in state |0〉, all we have is $U3\left(\frac{\theta }{2},0,0\right)$ followed by a $U3\left(\frac{-\theta }{2},0,0\right)$ and the effect is trivial. If the control qubit $|\Psi_{1}\rangle$ is in state |1〉, the net effect is a controlled rotation U3(θ,0,0) on the $|\Psi_{2}\rangle$ qubit.
- Measurement of the QSOD qubits to obtain the probabilities of alignment states.
  The measurements are mainly used in the end to extract computational results from the quantum states. This will allow for us to explore the quantum states of the qubits and make an interpretation that allows for improving the management system that is related to the industrial process.

## 4. Case Study. Qsod Circuit

We will propose a quantum circuit that allows calcullating the alignment states of the system that the state of the art based on genetic algorithms in order to illustrate the implementation of the QSOD method within a cyber-physical complex networked lean management system in an Industry 4.0 context. Following the recommendations of [56], we follow a clear case study roadmap to ensure the replicability and soundness of the results obtained. This roadmap has several phases:

- Section 4.1. Scope establishment.
- Section 4.2. Specification of population and sampling.
- Section 4.3. QSOD circuit design.
- Section 4.4. Analysis of results.

### 4.1. Scope Establishment

We aim to study the organizational alignment state of an Industry 4.0 factory that resembles a cyber-physical complex networked lean management system by modeling the strategic organizational design throughout a quantum circuit. The management networks that model such a system under study are based on the paradigm of formal standardized (CPD)nA communication among various leaders who follow the lean concept and represent value-creating processes that are driven by KPIs and their improvement of the system, as described in [39].

### 4.2. Specification of Population and Sampling

**Figure 3:**
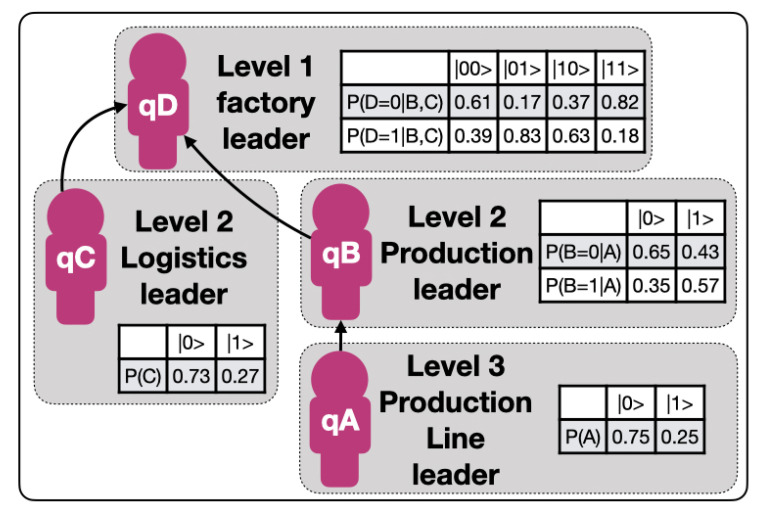
Case study.

Data on four team members in a factory at three relevant hierarchical levels are taken over twelve weeks daily: at level 1, the factory leader, at level 2, a logistics leader, and a production leader both reporting to level 1 and at level 3 a production line leader reporting to the level 2 production leader. Each leaders’ performance is measured through three KPIs, so there is a total of 12 KPIs being measured with the same frequency. The case study starts by creating a decision network from these data, as indicated in Section 3.2 and Figure 3. There are four nodes $q_{A}$, $q_{B}$, $q_{C}$, and $q_{D}$, which correspond to each process owner, and one ”dummy” node $q^{*}$, because the maximum number of parents is two. This network resembles the conditional probabilities of alignment and the respective dependencies related to the complex networked system provided by the original Hoshin Kanri Forest [10] complex network. The details of these calculations are omitted for clarity, since they are explained in the reference literature [2,9,10].

### 4.3. QSOD Circuit Design

We will now proceed to implement each of the indicated steps in Section 3.2 in a systematic way in order to generate a QSOD circuit that represents the alignment probabilities of the whole system. Each one of the 38 steps of the circuit has been denoted with a number in order to allow for a better understanding of the elements of the quantum circuit and facilitate the visualization of the effect of each step on the whole system

**Figure 4:**
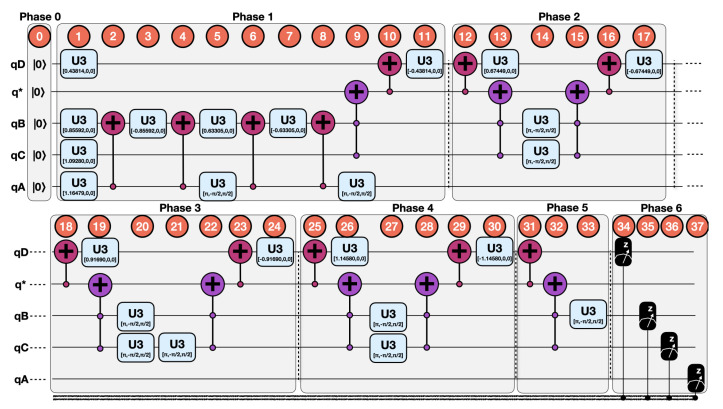
QSOD Circuit.

**Figure 5:**
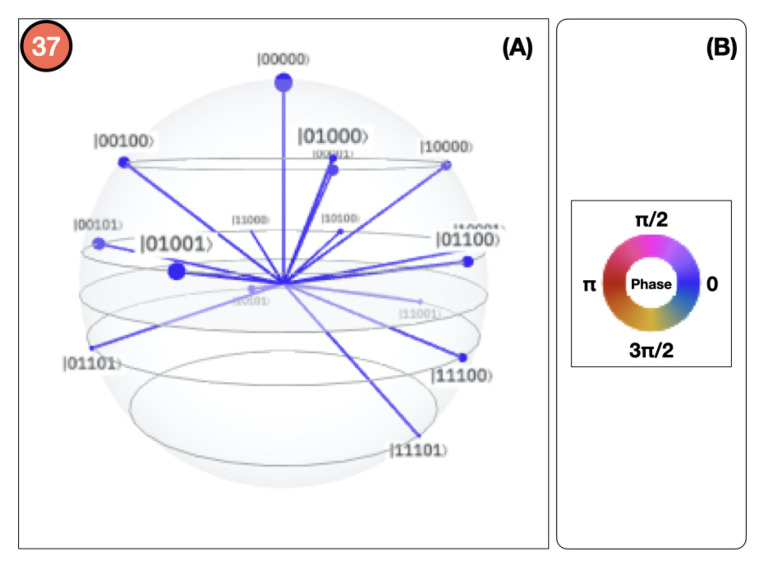
(**A**) QSOD Bloch sphere measurements of final state. (**B**) Phase state color code.

**Figure A1:**
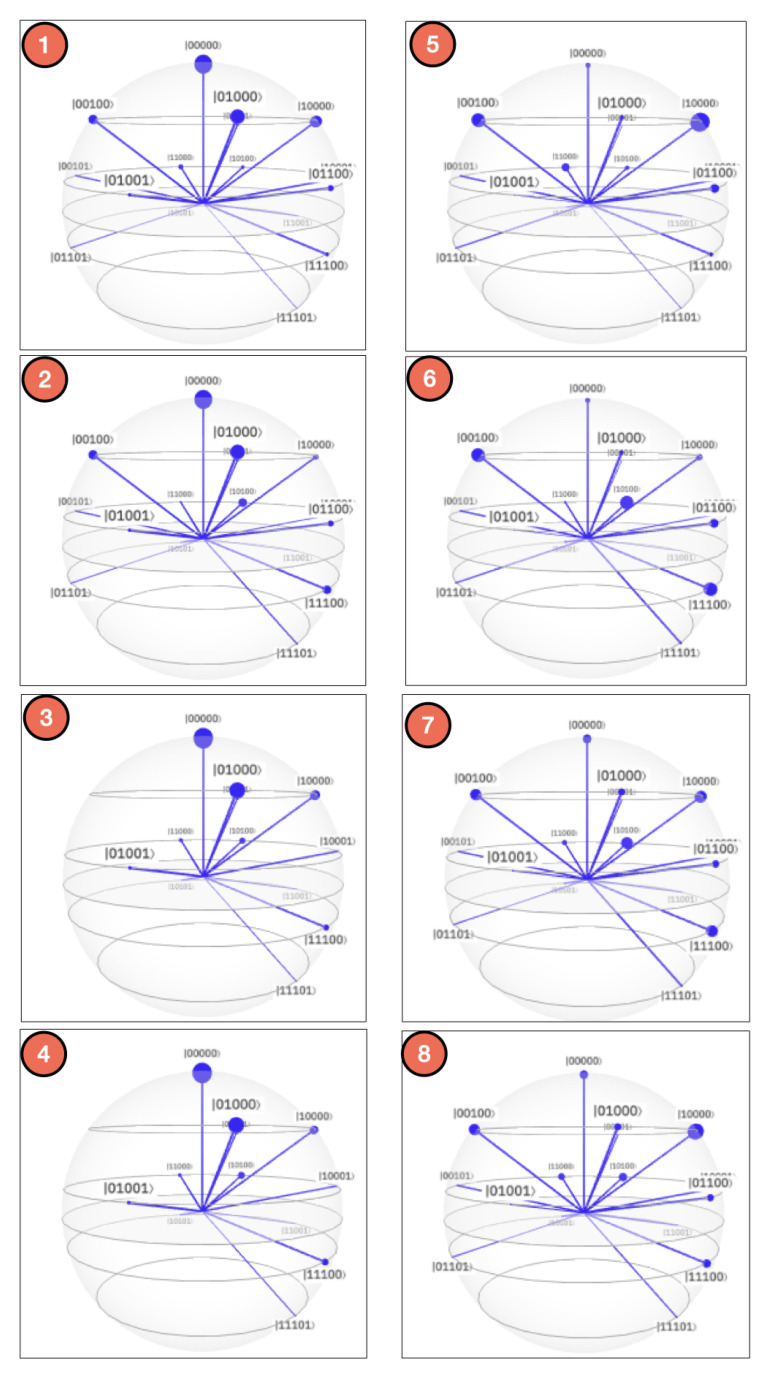
QSOD Bloch sphere measurements steps 1–8.

**Figure A2:**
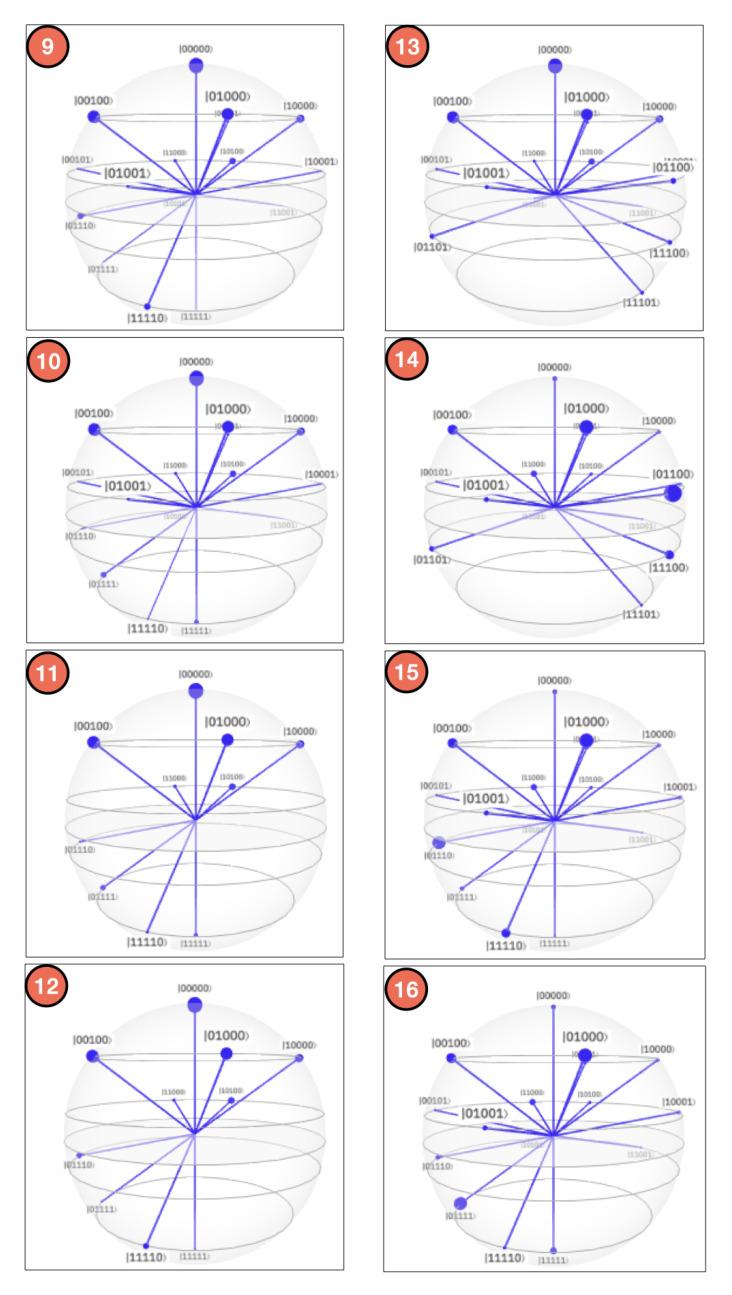
QSOD Bloch sphere measurements steps 9–16.

**Figure A3:**
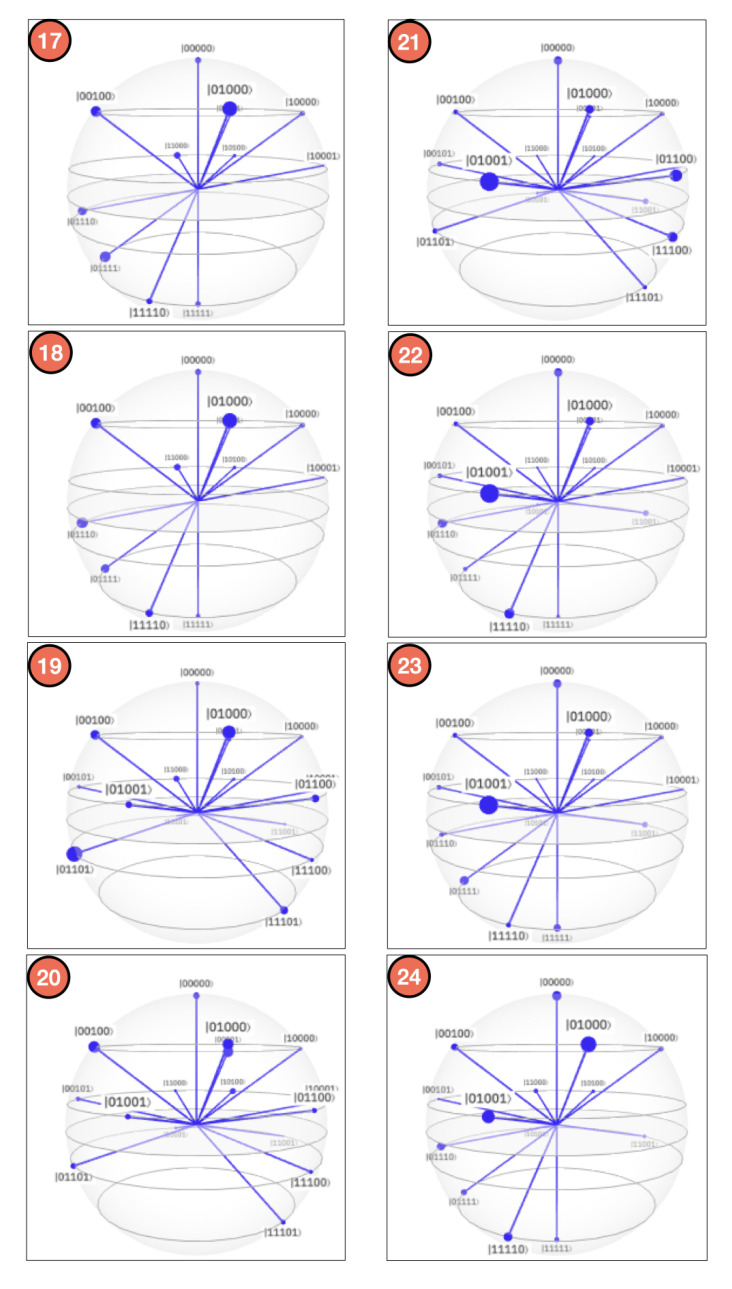
QSOD Bloch sphere measurements steps 17–24.

**Figure A4:**
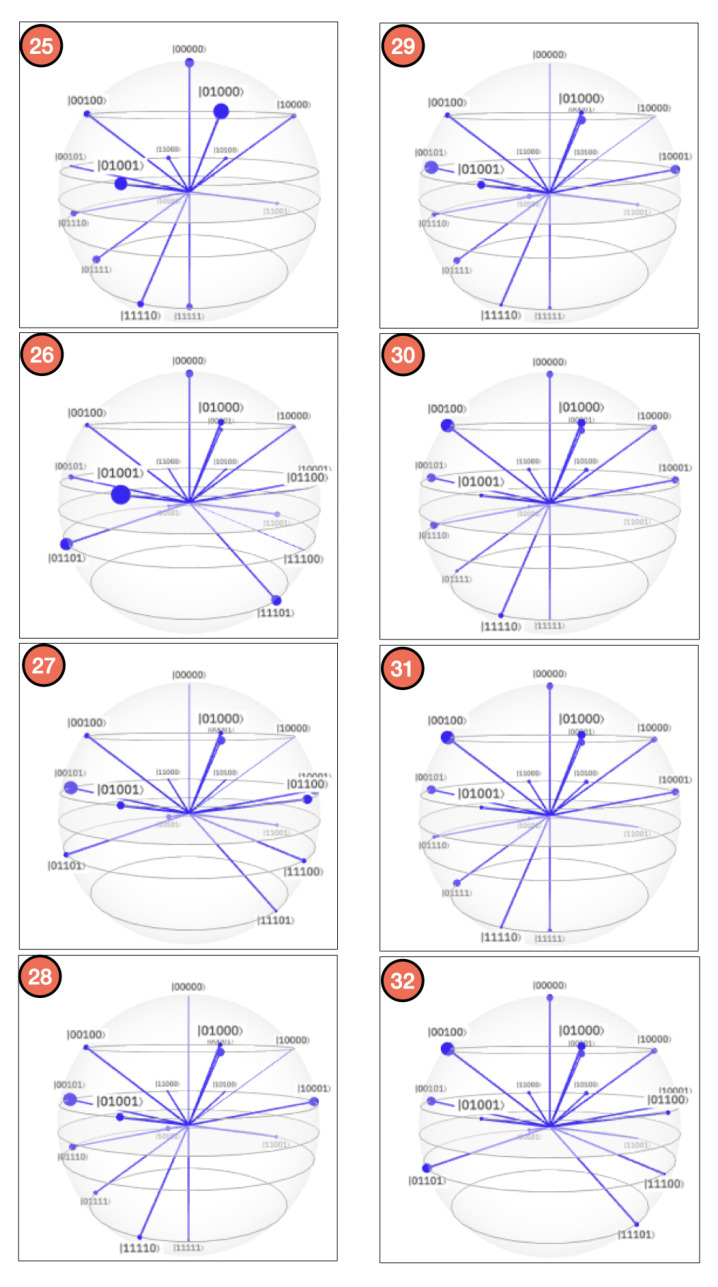
QSOD Bloch sphere measurements steps 25–32.

- Calculation of conditional probabilities.
  For each node, there are typically three related KPIs. The selected chromosome has subsequently 12 real numbers between 0 and 1, and we have used real value crossover and mutation with probabilities of 60% and 7%, respectively. The population was built over 8000 individuals and it ran over 1000 generations. Once the trajectories associated with each node have been calculated, it is trivial to calculate the relative probability of alignment or non-alignment at each node concerning those to which it is connected. This process is executed for each node in parallel without the loss of performance.
- Initialization and reset.
  In Step 0, each one of the nodes is assigned to a qubit, $q_{A}$, $q_{B}$, $q_{C}$, $q_{D}$, and a “dummy” node $q^{*}$ is created. The qubits are initialized and reset to |0〉 state. This allows for a controlled comparison of the probabilities through qubit rotations.
- Rotation angle computation.
  Following Equation (19) applied to each root qubit, we obtain the following results given by Equation (25):
  (25) $$\begin{array}{cc} \theta_{A}\overset{Equation\;(19)}{=}2\arctan\sqrt{\frac{0.25327658}{0.74672341}}=1.16479 & \theta_{C}\overset{Equation\;(19)}{=}2\arctan\sqrt{\frac{0.27}{0.73}}=1.0928 \end{array}$$
  Following Equation (20) applied to each child qubit, we obtain following results that are given by Equation (26):
  (26) $$\begin{array}{cc} \theta_{B,|0\rangle }\overset{Equation\;(20)}{=}2\arctan\sqrt{\frac{0.35}{0.65}}=1.2661 & \theta_{B,|1\rangle }\overset{Equation\;(20)}{=}2\arctan\sqrt{\frac{0.57}{0.43}}=1.7113\\ \theta_{D,|00\rangle }\overset{Equation\;(20)}{=}2\arctan\sqrt{\frac{0.39}{0.61}}=1.3489 & \theta_{D,|01\rangle }\overset{Equation\;(20)}{=}2\arctan\sqrt{\frac{0.83}{0.17}}=2.29161\\ \theta_{D,|10\rangle }\overset{Equation\;(20)}{=}2\arctan\sqrt{\frac{0.63}{0.37}}=1.83382 & \theta_{D,|11\rangle }\overset{Equation\;(20)}{=}2\arctan\sqrt{\frac{0.18}{0.82}}=0.87629 \end{array}$$
- Controlled rotations.
  By systematically applying Equation (21) to each qubit, we obtain the QSOD circuit that is shown in Figure 4.
  Figure 5A visualizes the quantum state of each step of the final state. The Bloch sphere provides a global view of a multi-qubit quantum state in the computational basis. Node size is proportional to state probabilities, and color reflects the phase of each basis state as shown in Figure 5B. The constant color blue that reflects the phase of each basis state does not change throughout the circuit. For the interested reader, in Appendix A, the Bloch sphere states of each step in the circuit are displayed in Figure A1, Figure A2, Figure A3 and Figure A4.
  The 37 steps that make up the QSOD have been divided into six distinct phases in order to improve the clarity of the explanation. We will now comment on the logic behind each of them:
  – Phase 0. Step 0.
    Phase 0 performs the initialization and reset, as previously explained in Section 4.3.
  – Phase 1. Step 1–Step 11.
    This phase is subdivided in three conceptual parts:
    First. This phase starts in Step 1 by rotating $q_{A}$ and $q_{C}$ by $\theta_{A}=1.16479$ and $\theta_{C}=1.0928$ radians respectively as calculated in Equation (25). This is because both $q_{A}$ and $q_{C}$ are root qubits and Equation (19) applies.
    Second, two controlled rotations on qubit $q_{B}$ in Steps 1–3 are performed, as calculated in Equation (26). As described in Equation (24), since qubit $q_{B}$ has a parent *qubit*$q_{A}$, we need to perform two controlled rotations properly aligned by CNOT gates. Depending on the state of qubit $q_{A}$: $\frac{\theta_{B,|1\rangle }}{2}=0.85592$ in Step 1 and $\frac{-\theta_{B,|1\rangle }}{2}=-0.85592$ in Step 3, in the case that $q_{A}$ is in state |1〉, and $\frac{\theta_{B,|0\rangle }}{2}=0.63305$ in Step 5 and $\frac{-\theta_{B,|0\rangle }}{2}=-0.63305$ in the case that $q_{A}$ is in state |0〉. In this case, a $U_{3}\left(\pi ,-\frac{\pi }{2},\frac{\pi }{2}\right)$ is performed, so as to generate proper alignment.
    Third, on qubit $q_{D}$, we need to perform a total of four controlled rotations throughout the circuit. This is because each of its parent qubits $q_{B}$ and $q_{C}$ can have two states, and we need to represent the rotations corresponding to the states |00〉, |10〉, |01〉, and |11〉. A controlled rotation $\theta_{D,|11\rangle }$ is performed on qubit $q_{D}$ conditioned by the state |11〉 of its parent qubits $q_{B}$ and $q_{C}$, as calculated in Equation (26). This is done with a controlled rotation $\frac{\theta_{D,|11\rangle }}{2}=0.43814$ in Step 1 and a controlled rotation $\frac{-\theta_{D,|11\rangle }}{2}=-0.43814$ in Step 11, properly aligned through CNOT and ccX gates, as described in Equation (21) in Steps 9–10 and Steps 31–33.
  – Phase 2. Step 12–Step 17.
    In phase 2, we perform the second controlled rotation on qubit $q_{D}$ as related to the states |00〉 of qubit $q_{B}$ and $q_{C}$. A controlled rotation $\theta_{D,|00\rangle }$ is performed on qubit $q_{D}$ conditioned by the state |00〉 of its parent qubits $q_{B}$ and $q_{C}$, as calculated in Equation (26). This is done with a controlled rotation $\frac{\theta_{D,|00\rangle }}{2}=0.67449$ in Step 13 and a controlled rotation $\frac{-\theta_{D,|00\rangle }}{2}=-0.67449$ in Step 17, properly aligned through CNOT and ccX gates, as described in Equation (21) in Steps 12 and Steps 14–16.
  – Phase 3. Step 18–Step 24.
    In phase 3, we perform the second controlled rotation on qubit $q_{D}$ as related to the states |10〉 of qubit $q_{B}$ and $q_{C}$. A controlled rotation $\theta_{D,|10\rangle }$ is performed on qubit $q_{D}$ conditioned by the state |10〉 of its parent qubits $q_{B}$ and $q_{C}$, as calculated in Equation (26). This is done with a controlled rotation $\frac{\theta_{D,|10\rangle }}{2}=0.91690$ in Step 19 and a controlled rotation $\frac{-\theta_{D,|10\rangle }}{2}=-0.91690$ in Step 24, properly aligned through CNOT and ccX gates, as described in Equation (21) in Steps 18 and Steps 20–23.
  – Phase 4. Step 25–Step 30.
    In phase 4, we perform the second controlled rotation on qubit $q_{D}$ as related to the states |01〉 of qubit $q_{B}$ and $q_{C}$. A controlled rotation $\theta_{D,|10\rangle }$ is performed on qubit $q_{D}$ conditioned by the state |01〉 of its parent qubits $q_{B}$ and $q_{C}$, as calculated in Equation (26). This is done with a controlled rotation $\frac{\theta_{D,|01\rangle }}{2}=1.1458$ in Step 26 and a controlled rotation $\frac{-\theta_{D,|01\rangle }}{2}=-1.1458$ in Step 30, properly aligned through CNOT and ccX gates, as described in Equation (21) in Steps 25 and Steps 27–29.
  – Phase 5. Step 31–Step 33.
    As mentioned earlier, in phase 5 the controlled rotation of qubit $q_{D}$ as related to the states |00〉 of qubit $q_{B}$ and $q_{C}$ started in Phase 1, qubits 1 and 9–11, is completed.
  – Phase 5. Step 34–Step 37.
    Finally, in phase 6 each one of the qubits is measured, as expressed by Equation (17).

### 4.4. Analysis of Results

The circuit is simulated on qiskit tool, a Python-based [57] quantum computing platform developed by IBM [58]. A total number of 8192 runs were carried out on the simulation with a total runtime of 3.8 s. In contrast, a genetic algorithm that would solve a similar problem with 12 KPIs (three per process owner) would take hundreds of hours for determining 48 real numbers between 0 and 1 in the chromosome with a value crossover and mutation with probabilities of 60% and 7%, respectively, with a population built over 8000 individuals and would run over 1000 generations. When compared with that, the performance increase of QSOD is remarkable. This has very powerful practical applications for industry leaders, since with this new approach they can potentially allow a better understanding of the complex processes underlying the strategic design of organizations and above all make decisions in real-time.

The obtained results are summarized in Table 1, which shows the total probability of each process owner $P_{j}\left(|0\rangle \right)$ to be in alignment and the reduced purity $\kappa_{j}$ of each *qubit* in the final state. We observe how the probability of alignment of the process owner D $P_{D}\left(|0\rangle \right)=49\%$, which indicates that the management system, as configured does not give a probability of achieving the alignment better than chance. That is why the probability of alignment of the root node representing process owner C $P_{C}\left(|0\rangle \right)=73\%$, the same as that presented in the decision network. The alignment probabilities representing process owners B $P_{B}\left(|0\rangle \right)=58.335\%$ and A $P_{A}\left(|0\rangle \right)=69.745\%$ are mixed probabilities. The nodes have a purity coefficient of over 90%, which indicates that there is almost no entanglement between them.

**Figure 6:**
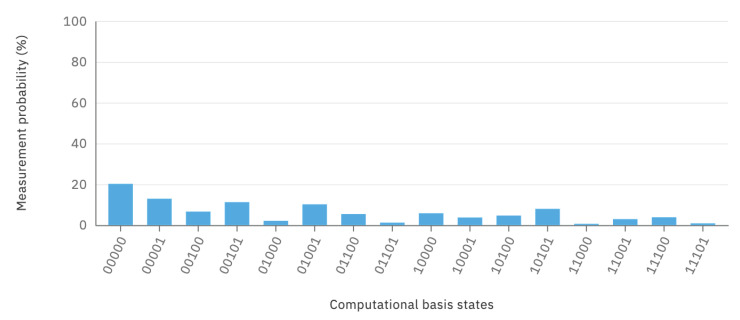
QSOD final Measurement Probabilities in step 37.

Further detailed results on the measurement probabilities of the computational basis states are visualized in Figure 6 after measurement Step 37. As an example of how to interpret these results, it can be said that the probability of complete system alignment, as described by the state |00000〉, is 20.18%.

## 5. Conclusions, Further Research and Limitations

This study shows how the strategic design of an organization is quantified through adequate modeling of decision networks expressed as quantum circuits. Because of the speed that quantum computing offers, the obtained results are promising, because they are likely to allow in the future a real-time simulation of organizational designs that could not have been done until now. This will allow for the leaders of the Industry 4.0, as well as interested scholars, to perform simulations of possible organizational designs for a customized adaptation of the strategic configuration. More specifically, an industrial leader will be able to potentially implement the algorithms that are presented to perform in-vitro analysis of certain clusters of interest within their organization, in order to make decisions on how the optimal configuration of their strategic industrial design should be. All of this in real-time and at virtually no cost. Presently, this tool can be used for a maximum of 15 *qubits* in the IBM simulation environments mentioned.

From systems engineering point of view, the proposed QSOD approach has great potential for supporting complex system development and management. Modern industrial enterprises consist of multiple systems, subsystems and even system of systems that usually involve different stakeholders with heterogeneous requirements. The alignment of these requirements is critical for decision-makings. A more specific example is the design and integration of modern manufacturing systems that might contain many digital twin models across the entire life-cycle of a product, such as product design, simulation, manufacturing, and maintenance, etc. The alignment of these systems is very challenging, even if not impossible, with traditional approaches due to limited computing resources and time. The proposed quantum strategic approach provides a promising solution for this challenge.

In addition to the applications in the management field, the proposed quantum-based approach could also be inspiring to technological domains such as the Distributed Ledger Technology (DLT), which has been widely applied in recent years. For example, DLT-based platforms have been developed in order to facilitate industrial data sharing, supply chain management and process monitoring etc. One of the main concerns about the most popular distributed ledger architecture blockchain is the vulnerability against quantum computing attacks. The proposed approach makes possible creating an assessment mechanism that is based on quantum computing principles in order to evaluate the security and robustness of distributed ledger applications, especially in the industrial domain.

For this work, we have used a classic computational tool that ideally simulates quantum circuits. This brings with it a series of potential restrictions to the study: on the one hand, it is possible to foresee a certain distortion when performing the same simulations in real quantum circuits. On the other hand, the number of qubits that the simulation can support is limited, at this moment of the state of the art, and therefore the simulation of large organizational networks still presents a challenge for future studies.

## Figures and Tables

**Table 1:** Summary of the results.

Qubits	Probability of Alignment $P_{j}\left(|0\rangle \right)$	Reduced Purity $\kappa_{j}$
$q_{D}$	49.135%	0.9178
$q_{B}$	58.335%	0.9026
$q_{C}$	73.000%	0.9178
$q_{A}$	69.745%	0.9794

## Abbreviations

The following abbreviations are used in this manuscript:

QSOD	Quantum Strategic Organizational Design
KPI	Key Performance Indicator
DLT	Distributed Ledger Technology

## Appendix A

This appendix shows, for the interested reader, the Bloch sphere states of each step in the circuit are displayed in Figure A1, Figure A2, Figure A3 and Figure A4. The states between steps 32 and 37 remain constant and are already represented in Figure 5A.
